# Supplementary material for: Identification of photoperiod-induced specific miRNAs in the adrenal glands of Sunite sheep (Ovis aries)
Source: Front Vet Sci. 2022 Jul 22;9:888207. doi: 10.3389/fvets.2022.888207 (PMC9354845; doi:10.3389/fvets.2022.888207)
Supplement: Supplementary file 1 [file Data_Sheet_1.ZIP › Supplementary file/miRDeep2 file.zip!/miRDeep2 file/Novel_Structure/SPLP42A2/Novel_217.pdf]

Provisional ID	:	NC_019467.2_20829
Score total	:	1.6
Score for star read(s)	:	-1.3
Score for read counts	:	0
Score for mfe	:	1.3
Score for randfold	:	1.6
Score for cons. seed	:	
Total read count	:	422
Mature read count	:	422
Loop read count	:	0
Star read count	:	0

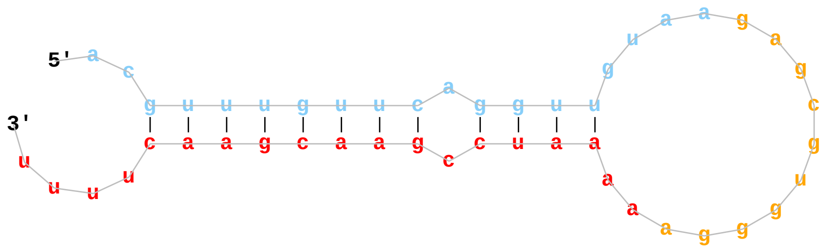

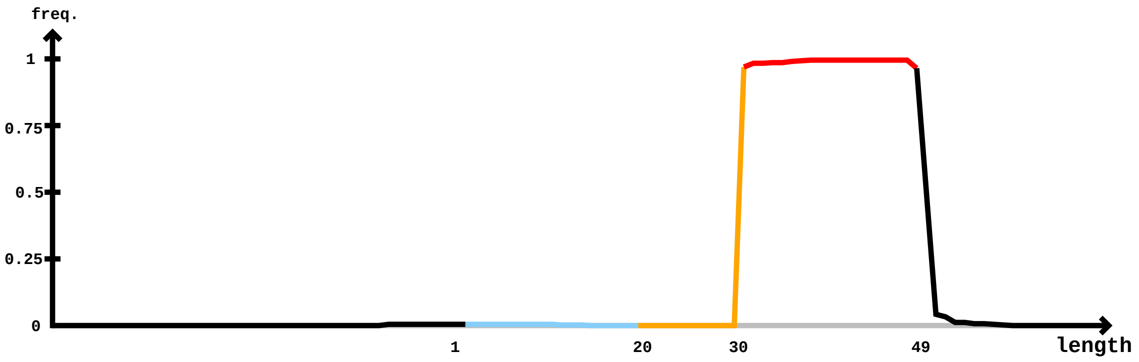


```
                                          Star                     Mature
5'- uggguucuguccuagguaaugguaguacuggguuggccaaaacguuuguucagguuguaagagcgugggaaaaauccgaacgaacuuuuuggccaagucaauaauuug   -3'   exp
    ..(((.(((.((........))))).)))((.((((((((((.((((((((.((((................)))).)))))))).)))))))))).)).........   reads    mm       sample
    .................................uggccaaaacguuuguuc.........................................................   1        0        seq
    .................................uggccaaaacguuuguucagg......................................................   1        0        seq
    ......................................................................aaaauccgaacgaacuuu....................   13       0        seq
    ......................................................................aaaauccgaacgaacuuuu...................   387      0        seq
    ......................................................................aaaauccgaacAaacuuuuu..................   3        1        seq
    ......................................................................aaaauccAaacgaacuuuuu..................   4        1        seq
    ......................................................................aaaauccgaacAaacuuuuug.................   3        1        seq
    ......................................................................aaaauccAaacgaacuuuuug.................   1        1        seq
    ......................................................................aaaauccgaacAaacuuuuugg................   1        1        seq
    ......................................................................aaaaucUgaacgaacuuuuugg................   2        1        seq
    .......................................................................aaauccgaacgaacuuuuugg................   6        0        seq
    .........................................................................auccgaacAaacuuuuuggcc..............   1        1        seq
    ...........................................................................ccgaacgaacuuuuuggccaa............   1        0        seq
    ...........................................................................ccgaacgaacuuuuuggccaaCu..........   1        1        seq
    ............................................................................cgaacgaacuuuuuggcc..............   1        0        seq
    .............................................................................gaacgaacuuuuuggccaag...........   1        0        seq
```
